# The association between problematic Internet use, eating disorder behaviors, and well-being among Palestinian university students

**DOI:** 10.1186/s41155-021-00198-5

**Published:** 2021-10-21

**Authors:** Fayez Mahmid, Dana Bdier, Priscilla Chou

**Affiliations:** 1 An-Najah National University, Nablus, Palestine; 2 Psychology and Counseling Department, An-Najah National University, Nablus, Palestine; 3 University of Guelph-Humber, Toronto, Canada

**Keywords:** Problematic Internet use, Eating disorder behaviors, Well-being, Palestinian university student*s*

## Abstract

**Objectives:**

The current study aimed to test the correlation between problematic Internet use, eating disorder behaviors, and well-being among Palestinian university students.

**Methods:**

To examine the relationship between the study variables, a correlational study was conducted. The geographical representation of the study sample showed that 48.1% of participants were from urban populations, 48.1% were from rural villages, and 3.8% were from internally displaced people’s camps.

**Results:**

Pearson’s correlation coefficient was used to test the relationship between problematic Internet use, eating disorder behaviors, and well-being. Results showed that problematic Internet use was negatively correlated to well-being (*r =* − .32*, p < .*01), and positively correlated to eating disorder behaviors *(r = .*39, *p <* .01*)*. The regression analysis found that problematic Internet use contributes statistically and significantly towards explaining variance in eating disorder behaviors (*B = .*46, SE *= .*08, *β* = .32). Moreover, well-being contributed in a way that was statistically significant towards explaining variance in eating disorders behaviors (*B =* − .39, SE *= .*09, *β* = − .25).

**Conclusion:**

The results of our study support previous studies that indicated that problematic Internet use was significantly and positively correlated with eating disorder behaviors, while it was significantly and negatively correlated to well-being among Palestinian university students. Further studies testing this relationship will be crucial in developing interventions to both reduce problematic Internet use and eating disorder behaviors and increase well-being among university students.

## Introduction

In recent years, Internet connectivity and technological advancement has increased drastically with more than 40% of the world’s population online (Ryding and Kaye, 2018). A report in 2012 found that children and adolescents in Australia spent nearly 24 h online per month with persons 18–24 years of age spending an average of 65 h online and individuals 25–34 years of age spending more than 100 h per month (Affouneh et al. 2021). In the Arab world, there are roughly around 135 million Internet users, with these staggering numbers it is evident that problematic internet use has emerged as a global concern (Khraim, 2016; Kuss et al. 2014; Poli, 2017).

Problematic Internet use can be defined as an over usage of the Internet; involving impairments in one’s psychological state, both emotionally and mentally, along with deficits in their scholastic, occupational and social interactions (Agbaria and Bdier, 2019). From a theoretical perspective, problematic Internet use can be a cause of both proximal and distal factors. Proximal causes include maladaptive cognitions that may reinforce an individual’s motivation to engage in compulsive Internet use (Griffiths et al. 2016). Distal causes can be pre-existing psychopathology (e.g., depression and social anxiety), and behavioral reinforcements from the Internet through rewarding cues that reinforce an individual’s conditioned responses (Agbaria and Bdier, 2019; Mahamid, Berte, and Bdier, 2021). Around 5 to 10% of Internet users can be considered Internet addicts with the majority of users are adolescents. For the youth, the Internet is considered as a main source of information, entertainment, and communication (Černja et al. 2019; Vuletić et al. 2014). The overall prevalence of Internet addiction in young adults in Bangladesh was 27.1%, addiction rates were 28.6% in the subgroup of 19–24 years and 23.5% in the subgroup of 25–35 years of age (Hassan et al. 2020). In a cross-sectional study, 49.5% of students at Jouf University in Saudi Arabia had moderate and severe Internet addictions (Abdel-Salam et al. 2019). Furthermore, an Italian survey among college students showed that 30% of students were problematic Internet users (Bianchini et al. 2017).

Palestinian youths and university students are at a greater vulnerability to develop behavioral problems such as Internet addiction. The youth have been experiencing several challenges and difficulties that are mainly characterized by environmental stressors (e.g., poverty, cultural pressures, militarization, lack of employment opportunities, future insecurity) and a lack of positive social outlets due to the restrictions on movement between communities, destruction of social networks, cultural standards of gender separation, and a lack of recreational facilities (Berte et al. 2019; Mahamid and Bdier, 2020a, b; Mahamid and Berte, 2019). Moreover, another expected challenge that Palestinian university students may face is the lack of mental health care institutions in Palestine especially at universities (Mahamid and Veronese, 2020; Marie et al. 2016).

Mahamid and Berte (2019) examined the notion of geopolitical vulnerability concerning occupation in a militarized area along with social media addiction in young adults. The findings demonstrated high rates of maladaptive social media use, the majority of university students received scores within the range of an addictive pattern of use (47%). Another study showed that 45.2% of Palestinian university students in the West Bank of Palestine received scores within the range of moderate social media addiction. Furthermore, 5.8% of individuals received scores in the category of severe/addicted Internet usage (Berte, Mahamid, and Affouneh, 2021). It is apparent that there is a vulnerability to the readily accessible and unrestricted usage of social networking and this could lead to maladaptive use to the detriment of one’s well-being.

### Problematic Internet use and eating disorder behaviors

Eating disorders can be defined as severe disturbances in body weight and eating behavior (Galmiche et al. 2019). It is a condition with manifestations in psychological and physical dimensions characterized by irregularities in the individual’s habits regarding eating, their body weight and appearance (Çelik et al. 2015). According to the Diagnostic and Statistical Manual of Mental Disorders (DSM-5), there are several forms of eating disorders including bulimia nervosa, anorexia nervosa, binge eating disorder, and related syndromes (APA, 2013). Additionally, eating behaviors reflect one’s relationship with food and behavioral responses to food cues within the environment (Russell and Russell, 2018).

Constant preoccupation with the Internet can contribute to the behavior of sedentarism as Internet addicts sit in front of their computers for an extended period, resulting in a lack of physical exercise (Bozkurt et al. 2018). This sedentarism may result in a neglect of consumption of healthy foods and increased ingestion of junk food due to its accessibility (Hinojo-Lucnena et al. 2019). Individuals can order food at home without having to spend a significant time cooking thus, remaining connected to the Internet. This can increase the risk of eating disorder behaviors such as dieting, binge-eating, and controlled eating (Oved et al. 2017; Said et al. 2018). Internet addiction is correlated with irregular sleep and this may result in weight gain (Mahamid, Berte, and Bdier, 2021). Many individuals today will find themselves scrolling mindlessly through social media, subconsciously viewing pictures of celebrities or others individuals who present an unrealistic beauty standard to the general public. Body dissatisfaction refers to negative views on one’s body leading to a perceived discrepancy between one’s assessment of one’s body as opposed to an ideal body (Kollei et al. 2017). Ubiquitous images of the ideal body have infiltrated the Internet promoting unattainable beauty standards through photo-shopped methods leading to bodily dissatisfaction (Liu et al., 2020). According to the objectification theory, constant body surveillance will result in anxiety and shame about one’s body, leading to mental health issues particularly, disordered eating (Ramos et al. 2019). This concern and dissatisfaction may manifest itself in an increased risk of eating disorders such as binge-eating, dieting, and loss of controlled eating (Hinojo-Lucena et al. 2019). Additionally, this increased access to the Internet had led to websites that encourage achieving low body weights promoting behaviors such as vomiting and fasting to obtain an ideal body (Borzekowski et al. 2010).

According to a study that targeted Palestinian university students, the prevalence of eating disorders is considerably high among female Palestinian university students (Saleh et al. 2018). Another study found that (38.9%) of females at Palestinian universities showed eating problem symptoms, an indication of the prevalence of eating disorders among Palestinian university students (Badrasawi and Zidan, 2019). Several studies demonstrated that the higher prevalence of eating disorders among Palestinian university students related to different psychosocial factors, such as political violence, media exposure, and problematic Internet use could be influencing youths’ attitudes related to body weight and body image (Badrasawi et al. 2021; Damiri et al. 2021; Radwan et al. 2021).

### Problematic Internet use and well-being

Given the proliferation of social networking websites and electronic devices, there is an increasing concern that this usage may harm the well-being of individuals. Well-being is the extent to which an individual is satisfied, happy, and enjoys their life. Subjective well-being can be characterized as how people experience the quality of their lives and appraise their internal experiences (Agbaria and Natur, 2018; Mahamid and Bdier, 2020b). Well-being is expected to be a protective factor against problematic Internet use among youths, as youths with high levels of well-being are able to use coping strategies when dealing with stressors, can engage in self-regulation, possess high levels of self-esteem and are less likely to be engaged in risky behaviors (Çakar and Tagay, 2017; Howard and Williams, 2018; Sagone and De Caroli, 2014).

Previous literature has indicated a negative relationship between problematic Internet use and well-being among university students. Individuals may be exposed to idealized depictions of others and thus, engage in upward comparisons resulting in decreased self-esteem (Schmuck et al. 2019). Additionally, Koç (2017) analyzed the relationship between subjective well-being and Internet addiction in university students, the results demonstrated that life satisfaction and positive feelings predicted a negative relationship with Internet addiction but negative feelings can positively predict Internet addiction. A significant amount of studies has found a correlation between high levels of Internet use in adolescence and poor mental health with an increase in depression, anxiety, self-harm, suicide ideation, attention deficit hyperactivity disorder, and hostility/aggression (Marchant et al. 2017; Carli et al. 2014). The prevalence of Internet usage may lead to the risk of cyber bullying which has been linked to adverse mental health outcomes such as self-harm, depression, suicide ideation, and anxiety (John et al. 2018; Fisher et al. 2016).

The geographic vulnerabilities of youth residing in Palestine are high. This is a result of a high level of restriction of movement due to cultural and geographical restraints (checkpoints, lack of reliable public transportation, and limited access of youth to private vehicles). There is a great amount of stress associated with the uncertainty of a highly volatile situation, which these youths have little control over. This lack of perceived sense of control can result in desires for escapism and greater vulnerability for addictive patterns with nearly 47% of Palestinian youth addicted to the Internet (Mahamid and Berte, 2019; Mahamid and Berte, 2020). Given the limited avenues of socialization and thus the default option of social networking and the possible ramification of Internet use impacting Palestinian youth’s eating behaviors, the following study examines this possible association.

### Current study

The current study was designed to test the association between problematic Internet use, eating disorders behaviors, and well-being among Palestinian university students. Based on previous research findings, the current study hypothesizes that (1) problematic Internet use is positively correlated with eating disorders among Palestinian university students, (2) well-being is negatively correlated with problematic Internet use among Palestinian university students, (3) and problematic Internet use is negatively correlated with well-being.

## Methodology

### Participants

The participants of the current study were recruited online via e-mails, social media, and SMS campaigns. The sample including 566 Palestinian university students, representing 260 females and 306 males. The geographic representation of the study sample indicated that 48.1% of participants were from an urban population, 48.1% were from rural villages, and 3.8% were from internally displaced people’s camps. In regard to education, 46.6% of participants had a graduate degree, and 53.4% had a bachelor’s degree. For inclusion in the study, participants were required to be (1) Palestinian, (2) native Arabic speakers, and (3) free from having been diagnosed with any form of neurodevelopmental or mental health impairment. Our study was approved by An-Najah Institutional Review Board (IRB) before data collection was administrated.

### Instruments and procedures

The instruments of our study were translated from English to Arabic and were reviewed for comprehensiveness and content validity. A pool of experts in clinical psychology, Arabic language, and psychological counselling reviewed the research instruments; minor modifications were made based on the reviewers’ feedback. An independent expert in the language translated the instruments into English. To test the reliability of the scales, fifty participants independent from the study sample were asked to answer the scales (reliability sample); Cronbach’s alpha indicated high internal consistency for the study instruments. The instruments of the study (General Well-being Scale [SGWB], The Problematic Internet Usage Scale [IDS9-SF], and The Eating Disorder Examination Questionnaire [EDE-Q]) were also validated by An-Najah IRB as they were used in previously published studies (Mahamid, Bdier, & Chou, 2021; Mahamid, Berte, and Bdier, 2021)

#### The Scale of General Well-being (SGWB)

The SGWB scale was designed by Longo, Coyne and Joseph (2018), the measure assesses well-being through fourteen specific constructs: happiness, vitality, calmness, optimism, involvement, self-awareness, self-acceptance, self-worth, competence, development, purpose, significance, self-congruence, and connection. The SGWB is a 5-point Likert scale offering five different options for the respondents. On average, means approximated the middle value of the 5-point format (i.e., 3). In our study, we tested the reliability of the scale using Cronbach’s alpha for internal consistency, and results of reliability indicated high internal consistency of the scale in the Palestinian context (0.90).

#### The Problematic Internet Usage Scale (IDS9-SF)

The IDS9-SF is a 9-item self-report scale designed by Pontes and Griffiths (2016) to test problematic Internet use. The scale is a 5-point Likert scale ranging from 1 (never) to 5 (very often). The total score of the scale is obtained by summing the scores of each item. The total score of IDS9-SF ranges from 9 to 45, with a higher score indicating a higher degree of problematic Internet use. In our study, we tested the reliability of the scale using Cronbach’s alpha for internal consistency, results of reliability indicated high internal consistency of the scale in the Palestinian context (.89).

#### The Eating Disorder Examination Questionnaire (EDE-Q)

The EDE-Q is a 28-item self-report scale designed by Fairburn and Beglin (1994) to test eating disordered behavior. The EDE-Q includes four subscales: restraint, eating concern, shape concern, and weight concern. The EDE-Q is a 7-point Likert scale offering seven different options for the respondents (0–6), with a higher score indicating a higher degree of an eating disorder. A total score EDE-Q is the sum of the four subscale scores divided by the number of subscales (i.e., four). In our study, we tested the reliability of the scale using Cronbach’s alpha for internal consistency, results of reliability indicated high internal consistency of the total scale in the Palestinian context (.90).

### Research procedures

Data collection was conducted in May 2021 and targeted Palestinian university students. The sample of our study was recruited using online sampling techniques. Participants of the study were provided with descriptions of the study instruments and the purpose of the study, which enabled them to make informed decisions as to whether or not they wanted to participate in the research. Participants who agreed to participate in the research signed informed consent. The study was approved by An-Najah National University Board (IRB). All procedures of our study were in line with the ethical guidelines of the American Psychological Association (APA, 2010) and the Declaration of Helsinki (1964).

### Data analysis

To test the descriptive statistics of problematic Internet use, eating disorder behaviors, and well-being; mean, standard deviations, percentages, range, minimum, max, skewness, kurtosis, and reliability analyses were performed. Moreover, Pearson’s correlation coefficient was used to evaluate the correlation between the aforementioned variables. Finally, regression analysis was conducted to assess the relationship between problematic Internet use, eating disorders, and well-being.

## Results

Descriptive statistics related to problematic Internet use, well-being, and eating disorder behaviors are shown in Table 1. Participants scored high on well-being, while moderate scores emerged on restraint eating, eating concerns, and the total score of eating disorders behaviors. Mild scores were prevalent with problematic Internet use, weight concerns, and shape concerns. Regarding internal consistency, all scales showed high reliability values ranging from .89 (shape concerns) to .93 (weight concerns).

**Table 1:** Means and standard deviations for research variables (*N* = 566)

Variable	Mean	S.D	Min	Max	Range	Skewness	Kurtosis	Reliability
Well-being	3.65	.03	1.64	5.00	3.36	− .19	− .08	.90
Problematic Internet use	1.97	.04	1.06	4.06	3.00	.89	.36	.89
Restraint eating	2.50	.06	.89	5.00	4.11	.87	− .23	.92
Eating concerns	2.47	.06	1.00	5.00	4.00	.94	− .10	.90
Weight concerns	1.97	.06	.75	5.00	4.25	1.03	.03	.93
Shape concern	1.80	.06	.29	5.00	4.71	1.42	1.25	.89
Eating disorder behaviors total	2.18	.06	.95	4.89	3.95	.98	.03	.90

Results of the correlational analysis are shown in Table 2. Specifically, well-being negatively correlated with problematic Internet use *(r =* − .32, *p < .*01*)*, restraint eating (*r =* − .27, *p < .*01), eating concerns *(r =* − *.*24, *p <* .01), weight concerns (*r* = − .35, *p* < .01), shape concerns (*r =*− .41, *p < .*01), and eating disorders total *(r =* − *.*35, *p < .*01*)*, while problematic Internet use positively correlated to restraint eating (*r =* .30, *p <* .01), eating concerns *(r* = .26, *p <* .01), weight concerns (*r* = .439, *p* < .01), shape concerns (*r = .*41, *p* < .01), and eating disorders behaviors total (*r* = .39, *p* < .01). Moreover, restraint eating positively correlated to eating concerns *(r = .92*, *p <* .01), weight concerns (*r* = .83, *p* < .01), shape concerns (*r = .*67, *p < .*01), and eating disorder behaviors total (*r* = .93, *p* < .01). Furthermore, eating concerns positively correlated to weight concerns (*r = .764, p <* .01), shape concerns (*r = .*58, *p < .*01), and eating disorder behaviors total *(r = .*58, *p < .*01*)*. Weight concerns positively correlated to shape concerns (*r = .*82, *p < .*01*)*, and eating disorders total (*r = .*93, *p < .*01). Finally, shape concerns positively correlated with eating disorder behaviors total (*r = .*88, *p <* .01).

**Table 2:** Correlations among study variables (*N* = 566)

Measures	(1)	(2)	(3)	(4)	(5)	(6)	(7)
(1) Well-being	1						
(2)Problematic Internet use	− .32**	1					
(3) Restraint eating	− .27**	.30**	1				
(4) Eating concerns	− .24**	.26**	.92**	1			
(5) Weight concerns	− .35**	.43**	.83**	.76**	1		
(6) Shape concerns	− .41**	.41**	.67**	.58**	.82**	1	
(7) Eating disorders behaviors total	− .35**	.39**	.93**	.87**	.93**	.88**	1

**p < 0.01

The results of regression analysis (Table 3) indicated that problematic Internet use statistically and significantly explained variance in eating disorders (*B = .*46, SE *= .*08, *β = .*32*)*. Moreover, well-being contributed significantly in explaining variance in eating disorders (*B =* − *.*39, SE *= .*09, *β =* − *.*25).

**Table 3:** Regression to predict eating disorder behaviors (*N* = 566)

Variable	B	SE	*Β*	t	p	95% CL
Gender	.21	.14	.08	1.46	.14	[− .07–.51]
Place	− .21	.13	− .04	− 1.37	.13	[− .48 to − .09]
Degree	− .06	.11	− .03	− .51	.60	[− .29–.16]
Problematic Internet use	.46	.08	.32	5.52	.00**	[.30–.63]
Well-being	− .39	.09	− .25	− 4.34	.00**	[− .58 to − .21]

**p < 0.01

## Discussion

The present study investigated problematic Internet use among Palestinian university students along with its relation to well-being and eating disorders. Additionally, associations between well-being and eating disorder behaviors were examined along with the relation between well-being and problematic Internet use. Problematic Internet use was linked to the presence of eating disorder behaviors in Palestinian university students and was found to influence individual’s well-being.

Our first hypothesis was confirmed, we found that problematic Internet use was positively correlated with Palestinian adolescent’s weight, eating, shape concerns about their body along with restraint eating thus, impacting the possibility of the development of an eating disorder. Each of these measures was correlated with the possible development of an eating disorder. Studies have found the risk for the development of eating disorders was greatest for individuals at risk for problematic Internet use attributed to a proliferation of weight loss websites and this idealization of thinspiration and/or fitspiration promoting eating disorder behaviors such as restrictive eating and extreme weight loss (Carrotte et al. 2015; Cataldo et al. 2020; Talbot et al. 2017). Our finding is in accordance with studies that have found that high Internet use was a significant predictor of eating attitudes among a sample of university students in Turkey (Çelik et al. 2015). Our results offer insight that high Internet use may have implications on behaviors surrounding eating and this could potentially lead to the development of an eating disorder. A study consisting of 12 studies for the systematic review and 10 studies for the meta-analysis revealed that problematic Internet use is a predictor of eating disorders in students in which different eating disorders: dieting, loss of controlled eating, food preoccupation, anorexia nervosa, bulimia nervosa, and binge-eating disorder (Hinojo-Lucena et al. 2019) were found to be related to problematic Internet use.

Our second hypothesis was also confirmed that well-being was negatively correlated with problematic Internet use in Palestinian university students. One’s well-being is an integral aspect of an individual’s soundness of body and mind (Alheneidi and Smith, 2020). Previous studies have also demonstrated that subjective well-being was negatively associated with problematic Internet use (Akin, 2012; Baggio et al. 2016). An explorative study conducted at An-Najah National University found that more than 47% of the students engaged in addictive patterns of Internet use (Mahamid and Berte, 2019). This heavy Internet usage can lead to exposure of idealized depiction and detrimental social comparison of others leading to envy (Krasnova et al. 2013), decreased self-esteem (Schmuck et al. 2019), and decreased perceived attractiveness (Peris et al. 2020). Additionally, problematic Internet use was found to be correlated with a decrease in life satisfaction and positive emotions (Sharma et al. 2020) with adolescents displaying affective temperament profiles and were found to be more emotional (Ozturk et al. 2013). The present study supports this line of reasoning as well.

The regression analyses revealed that Internet usage can explain the variance in eating disorders. Problematic Internet use in vulnerable populations can further complicate the issue of eating problems. Individuals living in the occupied territories of Palestine may be considered as vulnerable populations as they live under unique circumstances (e.g., militarization, poverty, lack of employment opportunities, cultural pressures). In this situation, it is likely that a vulnerability to the easily accessible and unrestricted social networks of social media could lead easily to excessive and maladaptive use in the face of heightened stressors and few alternative avenues for socialization, which may lead to eating disorders and mental health problems (Mahamid and Berte, 2019).

Our study demonstrated that Internet use can negatively correlate with one’s well-being. This finding reinforces other studies that have found that problematic Internet use has been found to be associated with dysfunctional coping strategies along with worse interpersonal relations critical for one’s overall physical and emotional happiness (Cudo et al. 2016). Studies have also revealed that individuals who engage in high Internet usage were more likely to possess low self-esteem, greater loneliness (Mei et al. 2016), greater depression, and anxiety scores (Li et al. 2017) and decreased self-efficacy (Berte et al. 2019). Spending an excessive amount of time online can be utilized as a coping mechanism against psychosocial problems. Their findings highlighted the link between problematic Internet use and poor psychological well-being with individuals with greater stress and lower self-esteem using the Internet to alleviate the hardships of everyday life (Kardefelt-Winther, 2014). The Internet can provide a medium for one to avoid coping with stressors through withdrawing, fantasizing, or rationalizing (Sela et al. 2019). Further, it can be argued that individuals with low self-esteem will face difficulty with controlling problematic Internet use behavior and comprehending the consequences associated with problematic Internet use (Mei et al. 2016). Studies have recommended that researchers should examine the need for improved self-control through emotional regulation or time management to reduce problematic Internet use (Hormes et al. 2014).

## Limitations

Our study has several limitations which may provide the opportunity for future studies targeting Palestinian university students. We utilized an online self-report method; this may have implications regarding validity as participants could have exaggerated their symptoms or under-reported the extent of their symptoms. Respondents could have been influenced to answer the questionnaire in a more socially acceptable manner. In order to generalize the findings, longitudinal and comparative investigations within different group of people are needed. Furthermore, the data was collected during the second COVID-19 lockdown in Palestine. The COVID-19 pandemic could have led to increased feelings of isolation and thus greater engagement with the Internet to stay connected, possibly skewing the results for the Internet questionnaire. More studies in the future are needed to generalize the findings of our study. Problematic Internet use, eating disorders, and well-being scales and their psychometric properties and factorial structure had not previously been tested in the Palestinian context within the Arabic language. More studies are needed to test the factorial structure and psychometric properties of these scales in the Palestinian context. Additionally, in our study we used correlational methods within a cross-sectional design. In order to make a causal interference based on the results of study, experimental and longitudinal studies should be conducted to test the correlation between these variables.

## Conclusion

The results of our current study supported previous studies indicating that problematic Internet use was positively associated with eating disorders, while well-being was found to be negatively associated with problematic Internet use and eating disorder behaviors. Both of these variables contributed significantly and statistically in explaining variance in eating disorder behaviors. The results of our study may contribute to explaining the theoretical correlation between study variables, it also provides practical implications through suggesting intervention studies targeting university students to reduce problematic Internet use and eating disorders behaviors and increase well-being during the critical life period of late adolescence. The results also reflect the need to enhance mental health services and educate the Palestinian university students about well-being and positive coping skills in order to better manage problematic Internet usage and eating problems during national and global health incidents. Recommendations include increasing general mental health resources (both access to clinics and public health messages) as well as those related specifically to mental health and subjective wellbeing during pandemics.

## Data Availability

The datasets generated during and/or analyzed during the current study are available from the corresponding author on reasonable request*.*
